# Commentary: Working toward a Multi-Program Strategy in Fall Prevention

**DOI:** 10.3389/fpubh.2017.00014

**Published:** 2017-02-13

**Authors:** Marcia G. Ory, Samuel D. Towne, Doris Howell, Cindy Quinn, Kelly J. Eblen, Suzanne M. Swierc, Matthew Lee Smith

**Affiliations:** ^1^College of Public Health, The University of Georgia, Athens, GA, USA; ^2^Texas A&M School of Public Health, Texas A&M University, College Station, TX, USA

**Keywords:** falls, fall prevention, fall prevention movement, coalitions, older adults

Falls among older adults are a critical public health issue, especially given the high rate of falls among older adults, the rapidly increasing number of older adults (both in the US and globally), and their substantial personal and societal costs (1, 2). In response, a national movement in the US toward a falls free society is underway (3, 4). According to Lynn Beattie’s commentary “Working toward a Multi-Program Strategy in Fall Prevention” (2015), “there is an inextricable link among aging processes, chronic diseases, and fall risks” (5). Yet, Beattie raises unanswered questions such as whether we can “consider a multi-program longer-term community strategy that helps to maintain behavior change, promotes physical activity, and helps to better manage medications and chronic conditions as a longer term fall prevention strategy.” This commentary reflects on a statewide strategy that considers risks, public health concerns, the structure and functioning of coalitions, and policy and programmatic impacts, and addresses Beattie’s question.

As illustrated in Figure 1, the major risks for falls and chronic conditions are often similar involving biological, behavioral, and environmental factors. While both falls and chronic conditions are interrelated and have similar roots, public health solutions are diverse in stakeholder engagement and strategies. For example, under the leadership of the National Council on Aging, there are state fall prevention coalitions in most (*n* = 46) states that promote and implement multilevel fall prevention strategies (6, 7). Similarly, the National Association of Chronic Disease Directors works through state and community partners to focus on solutions that help ameliorate chronic disease burden by addressing modifiable risk factors.^1^

**Figure 1:**
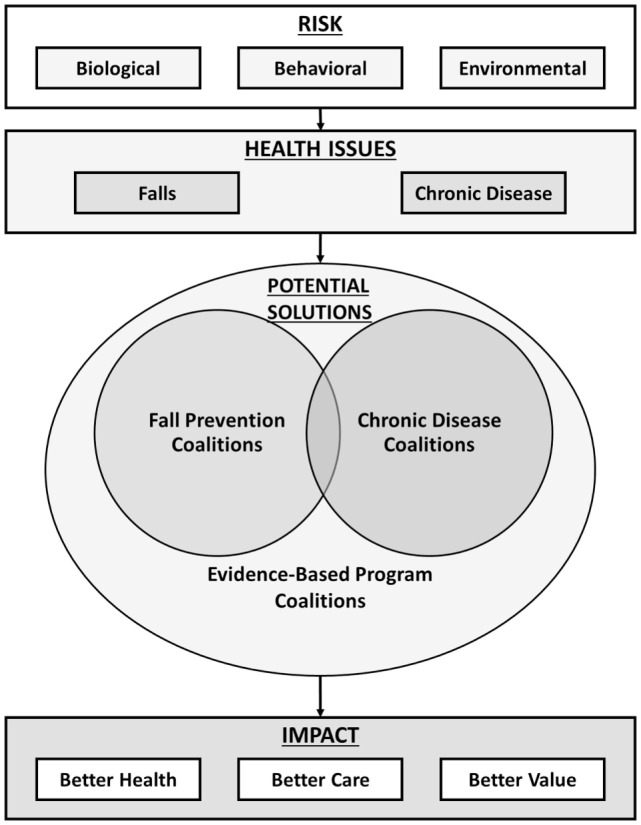
**Schematic overview of fall- and chronic disease-related risk, potential coalition-related solutions, and impacts**.

The Texas Falls Prevention Coalition (TFPC) was established in 2007 and revitalized in 2014 with new leadership at the Center for Population Health and Aging (8, 9). It consists of nearly 200 members driven by a steering committee of 14 professionals representing aging services, clinical practice and healthcare organizations, state government, and academia.^2^

Following the 2015 Falls Free^®^ National Falls Prevention Action Plan (10, 11), TFPC’s major goals are to:

- Increase awareness of the issue and effective prevention strategies.
- Increase provider participation in fall prevention practices.
- Increase the availability and accessibility of community programs and services.
- Enhance data surveillance collection, analysis, and systems linkages.
- Increase funding opportunities and investments for fall prevention.

The TPFC currently faces a series of questions in its endeavors to facilitate programs and policies that meet the Triple Aim of better health, better care, and better value (12). These are:

- Should fall prevention coalitions be charged with addressing chronic disease management and prevention?
- Should chronic disease prevention and management coalitions be established within states? Should these focus on specific diseases or should they be more general in nature? Should they be charged with addressing fall prevention?
- Should overarching evidence-based program coalitions be established to encompass falls and chronic diseases and coordinate all possible solutions for a multipurpose impact?

It is fortunate that there is an established infrastructure of state fall prevention coalitions across the US. This infrastructure encompasses a “menu” of evidence-based programs that focus on different topics and serve different purposes, often supported by the Administration for Community Living. States can choose many options to move forward to introduce and enhance long-term solutions for fall prevention and disease prevention/management. Despite the course of action, a community should consider underlying themes and recommendations for solutions. In Texas, TFPC stakeholders are actively investigating opportunities to guide and inform the selection of the most appropriate coalition-based solution(s). Our strategic planning processes and considerations are detailed below:

1. Consider the history-based perspective to identify previous successes and challenges. What resources and structures are already available in a particular state that can serve as a springboard for expanded activities?
2. Identify community-based perspectives. Who are the stakeholders who can help define what the community believes is important, feasible, and worth supporting?
3. Reach out to partners. What is the composition of the current partner networks and how can they purposively expand to advance community initiatives?
4. Bridge different community sectors. How can we best break down silos that hinder innovation, collaboration, and transparency to promote coordinated participant referrals to different evidence-based programs?
5. Develop programs and policies that can address both falls and chronic disease. How can fall prevention activities be integrated within more generic chronic disease prevention and management activities without losing the long-standing momentum achieved related to fall prevention?
6. Plan a strategy for scaling and sustaining fall prevention strategies. What actions are most effective for minimizing fall risks in large numbers of older adult and for embedding policies and programs into existing community and clinical infrastructures?

In considering these general themes, the TPFC recommends the following actions, which we recommend as best practices for other states wanting to meet the Triple Aim of healthcare reform. These include:

- Working with community stakeholders to identify policy and programmatic champions who can help build momentum for planned activities.
- Facilitating expanded partnerships among traditional aging services sectors, healthcare sectors, community-based organizations, and payers so that all can serve as an entry portal for health promotion and risk reduction.
- Bundling fall prevention and chronic disease management programs—employing health passports and other referral mechanisms to encourage older adults to sequentially transition from one health and wellness workshop to another.
- Examining program delivery patterns to develop strategies for enhancing the representativeness of populations and settings served.
- Creating an evidence-based program resource clearinghouse to assist in policy formation as well as program training and delivery.
- Utilizing tools such as the health savings cost estimator tool (13) for tracking costs and return on investment of different intervention strategies.^3^

The questions posed by Beattie (5) in her commentary continue to inspire and drive fall prevention efforts in Texas and across the US. These questions remind us that in the presence of challenge, there is need and opportunity for innovation. The potential solutions posed in Figure 1 provide options for employing coalitions to integrate fall prevention and chronic disease self-management approaches to improve the health and quality of life among older adults.

## Author Contributions

MO, ST, DH, CQ, KE, SS, and MS drafted, reviewed, and approved the manuscript.

## Conflict of Interest Statement

The authors declare that the research was conducted in the absence of any commercial or financial relationships that could be construed as a potential conflict of interest.

## Footnotes

^1^National Association of Chronic Disease Directors. (n.d.). Available from: http://www.chronicdisease.org.

^2^https://fallsfreetexas.org.

^3^http://www.ebp-savings.info.

## References

[1] Houry D Florence C Baldwin G Stevens J McClure R The CDC Injury Center’s response to the growing public health problem of falls among older adults. Am J Lifestyle Med (2016) 10(1):74–7. 10.1177/1559827615600137 PMC4681302 26688674

[2] Towne SD Jr Ory MG Smith ML Cost of fall-related hospitalizations among older adults: environmental comparison from the 2011 Texas Hospital Inpatient Discharge Data. Popul Health Manag (2014) 17(6):351–6. 10.1089/pop.2014.0002 25075812

[3] Ory MG Smith ML, editors. Evidence-Based Programming for Older Adults. Lausanne, Switzerland: Frontiers Media (2015).

[4] Schneider EC Beattie BL Building the older adult fall prevention movement-steps and lessons learned. Front Public Health (2016) 2:119 10.3389/fpubh.2014.00194 PMC4410413 25964916

[5] Beattie BL Working toward a multi-program strategy in fall prevention. Front Public Health (2015) 2:254 10.3389/fpubh.2014.00254 25964930 PMC4410622

[6] National Council on Aging. State Falls Prevention Coalition Contacts. Washington, DC: National Falls Prevention Resource Center, National Council on Aging, National Council on Aging (2016). Available from: https://www.ncoa.org/resources/list-of-state-falls-prevention-coalitions

[7] Schneider EC Smith ML Ory MG Altpeter M Beattie BL Scheirer MA State fall prevention coalitions as systems change agents: an emphasis on policy. Health Promot Pract (2015) 17(2):244–53. 10.1177/1524839915610317 26500227

[8] Ory MG Smith ML Wade A Mounce C Wilson A Parrish R. Implementing and disseminating an evidence-based program to prevent falls in older adults, Texas, 2007-2009. Prev Chronic Dis (2010) 7(6):A130. 20950537 PMC2995605

[9] Ory MG Smith ML Wade AF Wright JC Parrish R Addressing falls in Texas: evidence-based fall prevention programming for older adults. Texas Public Health Assoc J (2010) 62(1):15–20.

[10] National Council on Aging. 2015 Falls Free^®^ National Falls Prevention Action Plan. Washington, DC: National Falls Prevention Resource Center, National Council on Aging, National Council on Aging (2015). Available from: https://www.ncoa.org/resources/2015-falls-free-national-falls-prevention-action-plan

[11] Beattie BL Schneider ES State Policy Toolkit for Advancing Falls Prevention. Washington, DC: National Council on Aging (2012).

[12] Berwick DM Nolan TW Whittington J. The triple aim: care, health, and cost. Health Aff (2008) 27:759–69. 10.1377/hlthaff.27.3.759 18474969

[13] Ahn S Smith ML Altpeter M Post L Ory MG Healthcare Cost Savings Estimator Tool for Chronic Disease Self-Management Program (CDSMP): a new tool for program administrators and decision makers. Front Public Health (2015) 3:42 10.3389/fpubh.2015.00042 25964946 PMC4410329

